# A magnetic resonance imaging-based decision-making tool for predicting complex anal fistulas healing in the early postoperative period

**DOI:** 10.1186/s12876-023-02963-5

**Published:** 2023-10-31

**Authors:** Hao Xu, Guo-Zhong Xiao, Yi-Hui Zheng, Yuan-Ji Fu, Sheng-Lan Zhong, Dong-Lin Ren, Wen-Ru Li, Hong-Cheng Lin

**Affiliations:** 1 Department of Anorectal, Shanghai Fourth People’s Hospital, School of Medicine, Tongji University, Shanghai, 200434 China; 2 Department of Anorectal Surgery, Shuguang Hospital, Shanghai University of Traditional Chinese Medicine, Shanghai, 201203 China; 3 Department of Colorectal Surgery, The Sixth Affiliated Hospital, Sun Yat-sen University, Yuancun Er Heng Lu, No. 26, Guangzhou, 510655 China; 4 Guangdong Provincial Key Laboratory of Colorectal and Pelvic Floor Diseases, The Sixth Affiliated Hospital, Sun Yat-sen University, Guangzhou, 510655 China; 5 Guangdong Institute of Gastroenterology, Guangzhou, 510655 China; 6 Department of Radiology, The Sixth Affiliated Hospital, Sun Yat-sen University, Yuancun Er Heng Lu, No. 26, Guangzhou, 510655 China

**Keywords:** Anal fistulas, Magnetic resonance imaging, Nomogram, Prediction, Fistula healing

## Abstract

**Background:**

Magnetic resonance imaging (MRI) has excellent accuracy in diagnosing preoperative lesions before anal fistula surgery. However, MRI is not good in identifying early recurrent lesions and effective methods for quantitative assessment of fistula healing are still warranted. This retrospective study aimed to develop and validate a specific MRI-based nomogram model to predict fistula healing during the early postoperative period.

**Methods:**

Patients with complex cryptoglandular anal fistulas who underwent surgery between January 2017 and October 2020 were included in this study. MRI features and clinical parameters were analyzed using univariate and multivariate logistic regression analysis. A nomogram for predicting fistula healing was constructed and validated.

**Results:**

In total, 200 patients were included, of whom 186 (93%) were male, with a median age of 36 (18–65) years. Of the fistulas, 58.5% were classified as transsphincteric and 19.5% as suprasphincteric. The data were randomly divided into the training cohort and testing cohort at a ratio of 7:3. Logistic analysis revealed that CNR, ADC, alcohol intake history, and suprasphincteric fistula were significantly correlated with fistula healing. These four predictors were used to construct a predictive nomogram model in the training cohort. AUC was 0.880 and 0.847 for the training and testing cohorts, respectively. Moreover, the decision and calibration curves showed high coherence between the predicted and actual probabilities of fistula healing.

**Conclusions:**

We developed a predictive model and constructed a nomogram to predict fistula healing during the early postoperative period. This model showed good performance and may be clinically utilized for the management of anal fistulas.

## Background

Anal fistulas have a mean incidence of 8.6 cases per 100,000 of the population [1], with the surgical approach depending on the classification [2]. Magnetic resonance imaging (MRI) has been shown to be extremely accurate in identifying the perianal fistula anatomy, the presence and location of an internal opening, and the structural relationship between the fistula tract(s) and the main sphincter complex [3, 4]. The degree of accuracy has been improved by a range of techniques that routinely include T1- and T2-weighted imaging, and fat suppression sequences. Such examinations can qualitatively discriminate between the signal characteristics of active sepsis and those of fibrotic tissues. Consequently, the value of preoperative MRI in influencing the course of subsequent surgery has been well-documented, greatly reducing the likelihood of recurrence [5]. In addition to its crucial role in preoperative evaluation, MRI is highly accurate in identifying and diagnosing postoperative complications like abscess formation or tracts which were missed intraoperatively. After surgery, fistulas may recur even in apparently clinically healed tracts, where MRI can clearly detect recurrent or recrudescent abscess, even with external opening or openings have closed [6].

However, this MR-centered approach must consider the fact that in the immediate and early postoperative periods (before 12 weeks), an active fistula without abscess may be difficult to differentiate from healing granulation tissue. Both can initially appear hyperintense on T2W and STIR sequencing [6]. This adds many difficulties to clinical decisions regarding whether a relapse or a normal healing process occurs.

Two recent advances in MRI are diffusion-weighted imaging (DWI) and dynamic contrast-enhanced (DCE) sequences. These techniques provide an objective measure of diffusion and perfusion parameters associated with fistula activity [7]. Reflecting the limited diffusion of water molecules, DWI has a specific advantage in helps discriminate between an abscess and inflammatory mass [8]. In contrast, DCE-MRI permits the semiquantitative represent the signal intensity of the tissue [9], where in a previous study the absolute amounts of the time-intensity curves shape types correlate with the perianal disease activity index [10]. For most complex anal fistulas, improper management of the tracts and internal opening is one of the most common causes of fistula recurrence. As a result, early postoperative MRI evaluation helps to identify the presence of recurrence so that corrective measures can be promptly taken. Therefore, we aimed to assess the application of DWI and DCE in determining whether a relapse or normal healing process occurs at an early stage after fistula surgery. To our knowledge, many studies have used MRI parameters to build a nomogram model to predict the regression of patients with tumor [11–13]. Similar to these studies, the aim of our study was to explore and validate a specific MRI-based nomogram model to predict fistula healing during the early postoperative period. We suggest that this model would assist surgeons and patients in a shared decision-making process.

## Methods

### Study population

**Fig. 1:**
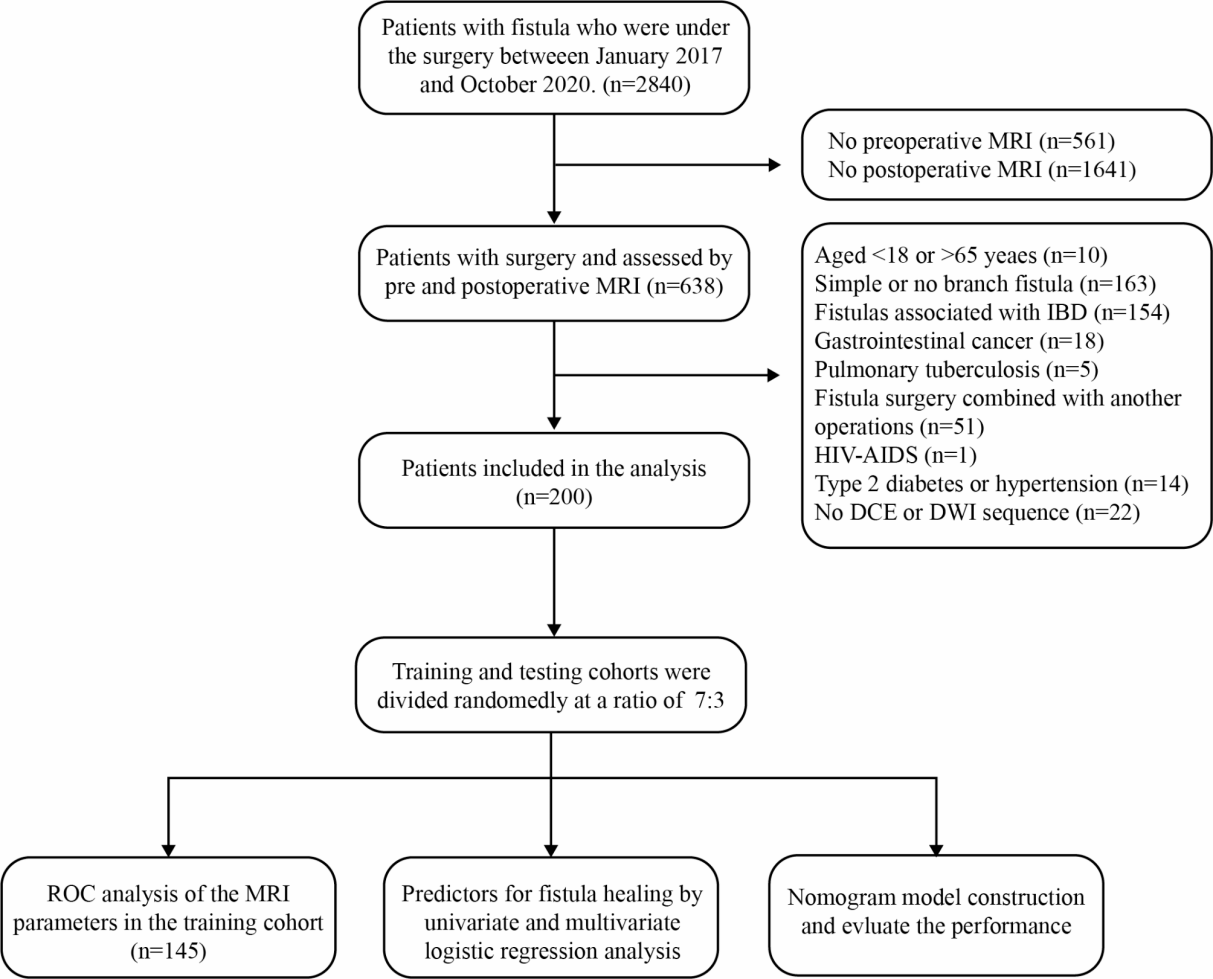
Flow chart of the study

This retrospective study was approved by Ethical Committee of the Sixth Affiliated Hospital of Sun Yat-Sen University (no. 2020ZSLYEC-199). The study database initially included data pertaining to 2,840 consecutive patients who were under the care of the Coloproctology Section of the Sixth Affiliated Hospital of Sun Yat-Sen University between January 2017 and October 2020. Based on the data presented in Fig. 1, it is evident that only approximately 22.5% of the reviewed patients had complete preoperative and postoperative MRI data. After the exclusion process, a total of 200 cases were ultimately included, all of which had complex cryptoglandular anal fistulas managed with a two-stage procedure and assessed using pre- and postoperative MRI examinations, enabling the use of their MRI data to develop a model for predicting fistula healing. Regarding the purposes of definition and analysis, complex cryptoglandular anal fistulas included high transsphincteric fistulas that exceeded one-third of the coronal length of the anal sphincter complex. They also included horseshoe anal fistula, supra- and extrasphincteric fistulas, fistula with lateral extensions, multiple tracts, and any case of translevator extension(s). The exclusion criteria were as follows: Patients aged < 18 or > 65 years, simple or no branch fistula, fistulas associated with inflammatory bowel disease (IBD), underlying gastrointestinal cancer, known pulmonary tuberculosis, fistula surgery combined with another operation, HIV-AIDS, type 2 diabetes or hypertension and no DCE or DWI sequence. The details are shown in Fig. 1.

### Clinical data collection

All patients in this study underwent preoperative MRI to assess the fistula anatomy. The patients included in this study were diagnosed with complex anal fistulas and underwent a two-stage surgical approach. In the first-staged surgery, a modified Henley procedure was performed with focus on curetting the branch tract(s) outside the EAS and placing a loose seton around the sphincters. Approximately one and half months later, the branch tracts were healed/nearly healed and another MRI examination was performed to identify whether the tract(s) outside the EAS healed. If the wound outside the EAS healed, the patients underwent a delayed second-stage procedure consisting of seton removal and fistulotomy. For a minority of patients, in outpatient practice a loose seton is tightened until falling out on its own.

Patient demographic data collected included age, sex, body mass index (BMI), fistula classification, alcohol intake history, smoking history and previous anal surgery history. Fistula healing was defined as complete epithelialization of the external opening without discharge [14, 15]. Alcohol intake was defined as current or prior habitual consumption (one to two times/week) of any amount or type of alcohol and then divided into two variables (drinking and no drinking) [16, 17]. Smoking was defined as self-reported smoking (smoking ≥ 5 cigarettes per day for > 6 months) [18].

### Quantitative MRI analysis

**Fig. 2:**
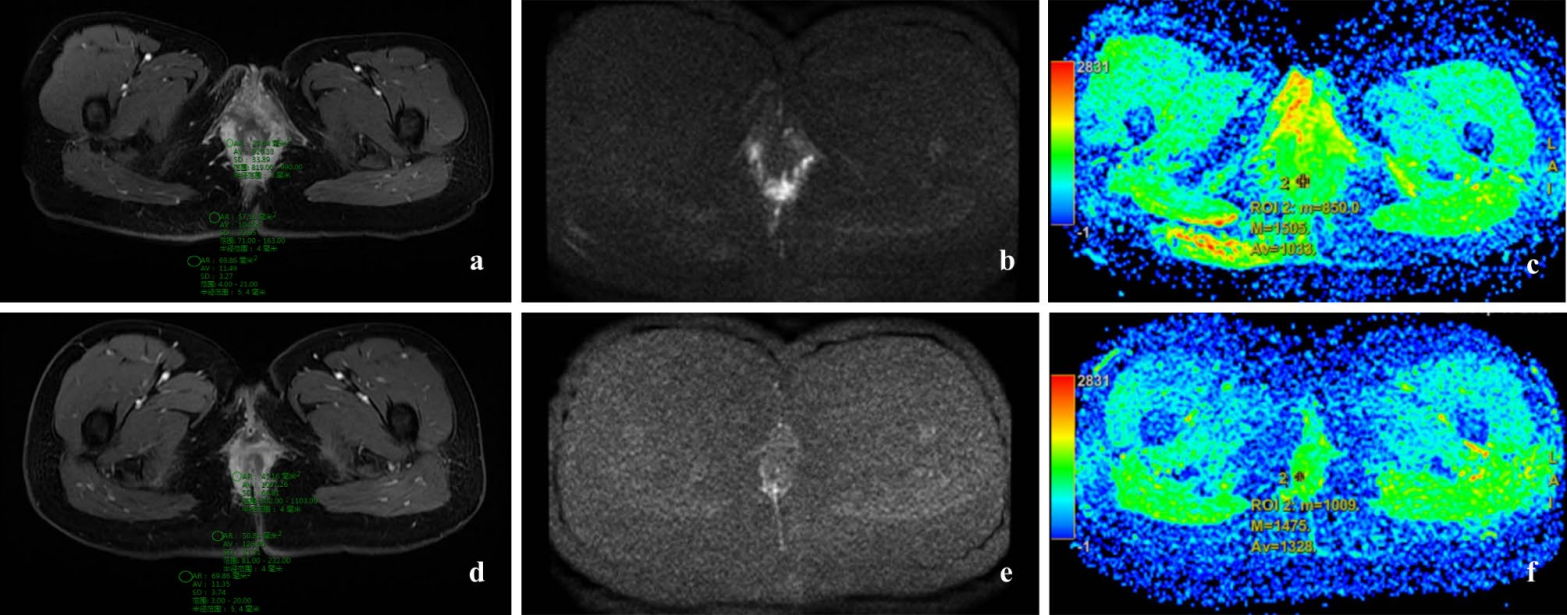
**a**. The fistula tracks visible on the preoperative DCE images, SI_lesion_, SI_tissue_, and SD_background_ are measured. **b**. In preoperative DWI in the axial plane (b = 1200), the fistula tracks have a high signal intensity. **c**. A ROI is manually positioned on the diffusion MR image and copied on the ADC map. **d**. The same level to measure the SI_lesion_, SI_tissue_, and SD_background_ on postoperative DCE images. **e**. Postoperative DWI (same level) in the axial plane (b = 1200), showing healed fistula tracks. **f**. A ROI is manually positioned on the diffusion MR image and copied on the ADC map. DWI, diffusion-weighted images; ROI, region of interest; MR, magnetic resonance; ADC, apparent diffusion coefficient; DCE, dynamic contrast-enhanced; SI, signal intensity

The MRI scans were evaluated by two doctors with extensive experience who were leading in interpreting pelvic MRIs. Consensus findings were recorded by a senior radiologist who were blinded to the clinical history and reference standard of the outcomes. We used T2-weighted and fat suppression sequences to reveal the anatomic structures of the anal region and fistula. For DWI, we delineated the region of interest (ROI) for the lesion area (b = 1200 s/mm^2^). They then located this region on an apparent diffusion coefficient (ADC) map. ADC values were calculated automatically on a dedicated workstation, as shown in Fig. 2 with measurements repeated thrice to obtain an average recording. Regarding DCE sequences, the contrast-to-noise ratio (CNR) in the Ax-LAVA Flex + C series was also measured thrice, and the average value was calculated as follows:

$$\small \small \small \small \small \normalsize  \ {\rm{CNR}}\,{\rm{ = }}\,\frac{{{\rm{|S}}{{\rm{I}}_{{\rm{lesion}}}} - {\rm{S}}{{\rm{I}}_{{\rm{normal}}\,{\rm{tissue|}}}}}}{{{\rm{S}}{{\rm{D}}_{{\rm{background}}}}}}$$

In this fomula, SI_lesion_ represents the fistula signal intensity (SI), whereas SI_normal tissue_ represents the SI of normal muscles around the fistulas. SD_background_ reflects the standard deviation of background signal [19].

### Statistical analysis

Continuous data are presented as medians and ranges, while categorical variables are expressed as numbers and percentages (%). The Mann-Whitney U test was used to compare continuous data, and the Chi-square or Fisher’s exact test was used where appropriate for comparisons between categorical variables. Receiver operating characteristic (ROC) analysis was performed to define the optimal cut-off values for ADC and CNR by calculating the area under the curve (AUC), other indicators include sensitivity and specificity. Risk factors for fistula healing were assessed using univariate analysis, and significant variables (*P* < 0.1) were incorporated into a multivariate logistic regression analysis. Factors with prognostic significance in the multivariate logistic regression analysis were used to build a fistula-healing model. A nomogram was used to visualize the model. Prediction models were developed with a random sample of 70% of the cohort as the training cohort, and then validated with the remaining 30% of the cohort as the validation cohort. Decision curve analysis and calibration curves were used to evaluate the model’s predictive performance. Statistical analyses were performed using R software version 4.1.0. *P* values < 0.05 were considered significant.

## Results

### Clinical characteristics and basic MRI features of the patients

The baseline clinical characteristics of the patients are summarized in Table 1. A total of 200 patients were included in the study, of whom 186 (93%) were male. The median age and BMI were 36 years (range: 18–65 years) and 23.8 kg/m^2^ (16.9–35.9 kg/m^2^), respectively. A total of 92 (46%) patients had a smoking history and 28 (14%) had a history of alcohol intake. Seventy one (35.5%) and 109 (54.5%) patients had recurrent and horseshoe fistulas, respectively. In total, there was a 19.5% incidence of suprasphincteric fistula in this cohort.The median time from preoperative MRI examination to the first stage surgery were 3 days (range: 0–39 days). The median time from the first stage surgery to postoperative MRI examination were 62 days (range: 26–179 days). The median postoperative CNR values of healed fistulas were lower than those measured in non-healed cases (36.6 *versus* 99.9; *P* < 0.01). The median postoperative ADC values of healed fistulas were higher than those measured in non-healed cases (1.35 × 10^− 3^ mm^2^/s *versus* 1.28 × 10^− 3^ mm^2^/s; *P* < 0.05). In total, there were no significant differences between the two groups except for alcohol intake history, postoperative CNR value and postoperative ADC value.

**Table 1:** Clinical characteristics of the patients (n = 200)

Patient characteristic no. (%) / median(range)	Healing (n = 101)	Non-healing(n = 99)	*P*
Male (%)	92(91.1)	94(94.9)	0.43
Age (years)	37(19 ~ 65)	34(18 ~ 61)	0.33
BMI (Kg/m^2^)	23.7(17.9 ~ 35.9)	23.9(16.9 ~ 34.6)	0.6
Smoking history (%)	40(39.6)	52(52.5)	0.09
Alcohol intake history (%)	8(7.9)	20(20.2)	0.02
Previous anal surgery (%)	70(69.3)	64(64.6)	0.58
Fistula classification (%)			0.41
Intersphincteric	23(22.8)	19(19.2)	
Transsphincteric	62(61.4)	55(55.6)	
Suprasphincteric	15(14.8)	24(24.2)	
Extrasphincteric	1(1)	1(1)	
Horseshoe fistula (%)	55(54.5)	54(54.5)	1.00
Recurrent fistula (%)	38(37.6)	33(33.3)	0.63
Postoperative CNR value	36.6(2.1 ~ 156.3)	99.9(13.9 ~ 514.1)	< 0.01
Postoperative ADC value (10^− 3^mm^2^/s)	1.35(0.2 ~ 2.08)	1.28(0.82 ~ 2.01)	0.01

### Predictors for fistula healing in the training cohort

**Fig. 3:**
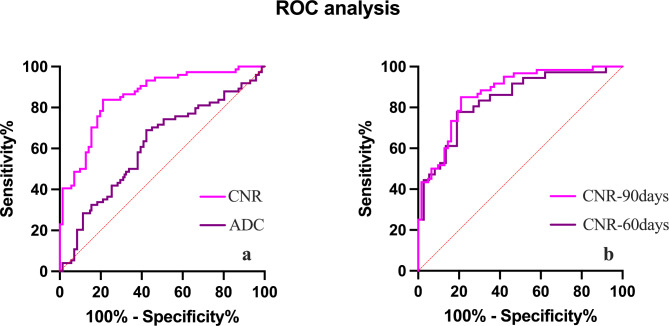
**a**. ROC curve for optimal cut-off levels of CNR and ADC values. **b**. ROC curve for optimal cut-off levels of 60 and 90 days CNR values. ROC, receiver operating characteristic curve; CNR, contrast-to-noise ratio; ADC, apparent diffusion coefficient

The data were randomly divided into the training and testing cohort at a ratio of 7:3. The characteristics of the patients in the training and testing cohort are displayed in Table 2. To explore the cut-off value of the CNR and ADC, we performed ROC analysis in the training cohort. Based upon the ROC analysis, the optimal CNR cut-off for healing fistula was 63.51 with an AUC of 0.854. This provided a sensitivity of 83.8% and a specificity of 78.9%. Additionally, an optimal ADC cut-off value for a healing fistula, which was 1.34 × 10^− 3^ mm^2^/s with an AUC of 0.612, provided a sensitivity of 68.9%, specificity of 57.7% (Fig. 3a). Given the performance characteristics of the CNR, we demonstrated its ability to predict early fistula healing using ROC analysis. The AUC value was 0.837 in the 60-day period and 0.868 in the 90-day period by ROC analysis. In the 60 days therapeutic range, a CNR cut-off of 65.83 yielded a sensitivity of 77.8%, specificity of 81.1%. In addition, in the 90 days therapeutic range, a CNR cut-off of 65.33 resulted in a sensitivity of 85.0%, specificity of 79.0% (Fig. 3b).

**Table 2:** Clinical characteristics of patients in training and testing cohorts (n = 200)

Patient characteristic no. (%) / median(range)	Training cohort			Testing cohort		
	Healing (n = 71)	Non-healing (n = 74)	*P*	Healing (n = 30)	Non-healing (n = 25)	*P*
Male (%)	67(94.4)	70(94.6)	1	25 (83.3)	24 (96.0)	0.29
Age (years)	37(19–65)	34(18–61)	0.52	36(25–61)	36(19–49)	0.53
BMI (Kg/m^2^)	23.7 (18.5–35.9)	23.4 (16.9–32.1)	0.10	23.6 (17.9–30.9)	25.5 (18.9–34.6)	0.07
Smoking history (%)	30(42.3)	41(55.4)	0.16	10 (33.3)	11 (44.0)	0.59
Alcohol intake history (%)	6(8.5)	14(18.9)	0.11	2(6.7)	6 (24.0)	0.15
Previous anal surgery (%)	49(69.0)	44(59.5)	0.30	21 (70.0)	20 (80.0)	0.59
Fistula Classification (%)			0.34			0.54
Intersphincteric	20(28.2)	15 (20.3)		3 (10.0)	4 (16.0)	
Transsphincteric	42 (59.2)	42 (56.8)		20 (66.7)	13 (52.0)	
Suprasphincteric	8 (11.3)	16 (21.6)		7 (23.3)	8 (32.0)	
Extrasphincteric	1(1.4)	1 (1.4)		0(0)	0(0)	
Horseshoe fistula (%)	37 (52.1)	41 (55.4)	0.82	18 (60.0)	13 (52.0)	0.75
Recurrent fistula (%)	29 (40.8)	21 (28.4)	0.16	9 (30.0)	12 (48.0)	0.28
Postoperative CNR value	36.6 (6.3-156.3)	93.6 (13.9-384.1)	< 0.01	39.6 (2.1-119.5)	103.6 (55.2-514.1)	< 0.01
Postoperative ADC value (10^− 3^mm^2^/s)	1.4 (0.2–2.1)	1.3 (0.8-2.0)	0.02	1.3 (1.0-1.6)	1.2 (0.9–1.7)	0.17

Subsequently, logistic regression was performed in the training cohort to develop the predictive model. Data regarding sex, age, alcohol intake history, smoking history, BMI, recurrent fistula, horseshoe fistula, fistula classification (Intersphincteric fistula, transsphincteric fistula, suprasphincteric fistula and extrasphincteric fistula), CNR value, and ADC value were considered potential prognostic factors affecting fistula healing and were included in the univariate analysis. The results suggested that alcohol intake history (*P* = 0.07), suprasphincteric fistula (*P* = 0.08), CNR value (*P* < 0.01), and ADC value (*P* < 0.01) were potential independent factors for fistula healing. These factors were then fitted in the multivariate analysis, which showed that no alcohol intake history (odds ratio [OR] = 4.46, 95% confidence interval [CI]: 1.16–17.17, *P* = 0.03), suprasphincteric fistula (OR = 0.17, 95% CI: 0.04–0.78, *P* = 0.02), CNR value (OR = 0.04, 95% CI: 0.01–0.1, *P* < 0. 01) and ADC value (OR = 2.78, 95% CI: 1.12–6.89, *P* = 0.03) were significantly correlated with fistula healing (Table 3).

**Table 3:** Univariate and Multivariate analysis of factors associated with fistula healing in the training cohort

Factors	Univariate analysis			Multivariate analysis		
	OR	95%CI	*P*	OR	95%CI	*P*
Sex (female/ male)	1.04	0.25–4.35	0.95	-	-	-
Age (years) (≤ 40/>40)	1.36	0.68–2.70	0.38	-	-	-
BMI(Kg/m^2^)						
>23.9	-	-	-	-	-	-
18.5–23.9	1.02	0.53–1.99	0.95	-	-	-
<18.5	-	-	0.99	-	-	-
Smoking history(no/yes)	1.7	0.88–3.28	0.11	-	-	-
Alcohol intake history(no/yes)	2.53	0.91-7	0.07	4.46	1.16–17.17	0.03
Previous anal surgery(no/yes)	0.66	0.33–1.31	0.23	-	-	-
Classification						
Intersphincteric	-	-	-	-	-	-
Transsphincteric	0.75	0.34–1.66	0.48	0.76	0.26–2.21	0.62
Suprasphincteric	0.38	0.13–1.11	0.08	0.17	0.04–0.78	0.02
Extrasphincteric	0.75	0.04–12.99	0.84	0.45	0-44.35	0.73
Horseshoe fistula (no/yes)	1.14	0.59–2.19	0.69	-	-	-
Recurrent fistula (no/yes)	0.57	0.29–1.15	0.12	-	-	-
CNR (> 63.51/≤63.51)	0.05	0.02–0.12	< 0.01	0.04	0.01–0.1	< 0.01
ADC (10^− 3^mm^2^/s) (> 1.34/≤1.34)	2.86	1.45–5.65	< 0.01	2.78	1.12–6.89	0.03

### Nomogram construction

**Fig. 4:**
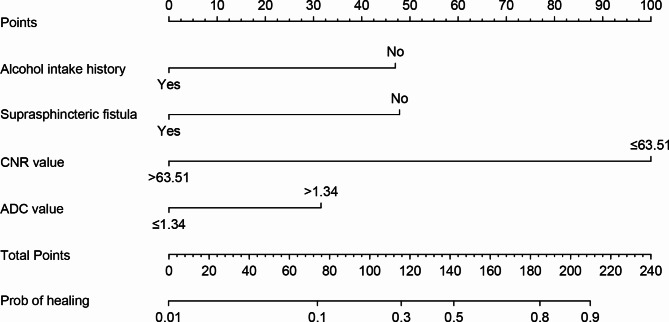
The nomogram to predict fistula healing by four independent prognostic factors. (To use the nomogram, an individual patient’s value is located on each variable axis, and a line is drawn upward to determine the number of points received for each variable value. The sum of these numbers is located on the Total Points axis, and a line is drawn downward to the healing axes to determine the likelihood of healing.)

Based on the results of multivariate analysis four independent prognostic factors (alcohol intake history, suprasphincter fistula, CNR value and ADC value) were incorporated to establish a predictive model for fistula healing (Fig. 4). This model was visualized using a nomogram, the usage of which was illustrated with an assumptive patient after surgery for an anal fistula, who did not have a history of alcohol intake, CNR value ≤ 63.51, ADC value > 1.34 × 10^− 3^ mm^2^/s and was a suprasphincteric fistula. The number of points for history of alcohol intake, CNR value, ADC value and suprasphincteric fistula was 47, 100, 31, and 0, respectively. A total of 178 points were added to this patient, which represented an approximately 75% chance of the fistula healing.

### Performance of the nomogram in the training and testing cohorts

**Fig. 5:**
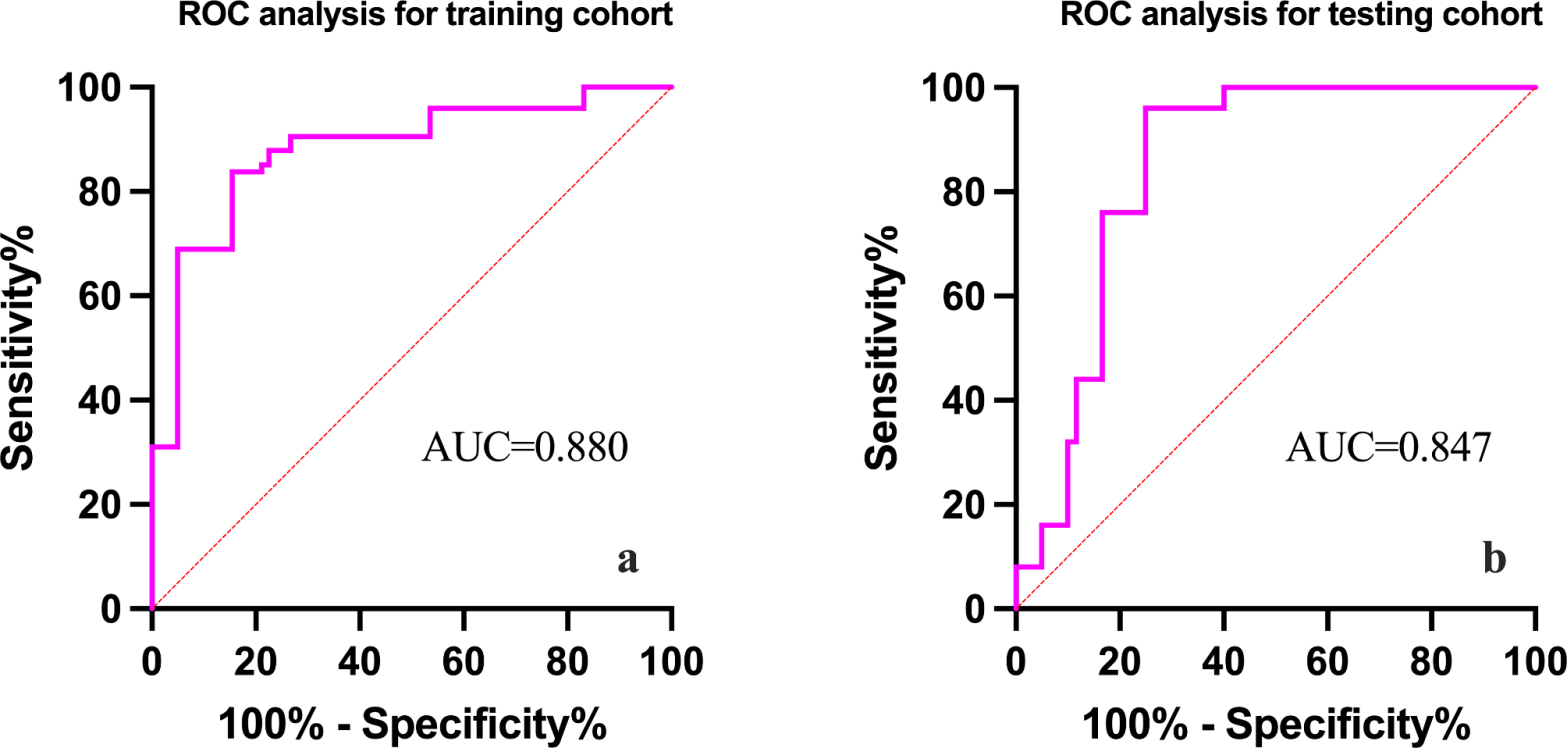
ROC analysis to predict fistula healing. **a**. ROC curve in the training cohort. **b**. ROC curve in the testing cohort. ROC, receiver operating characteristic curve

**Fig. 6:**
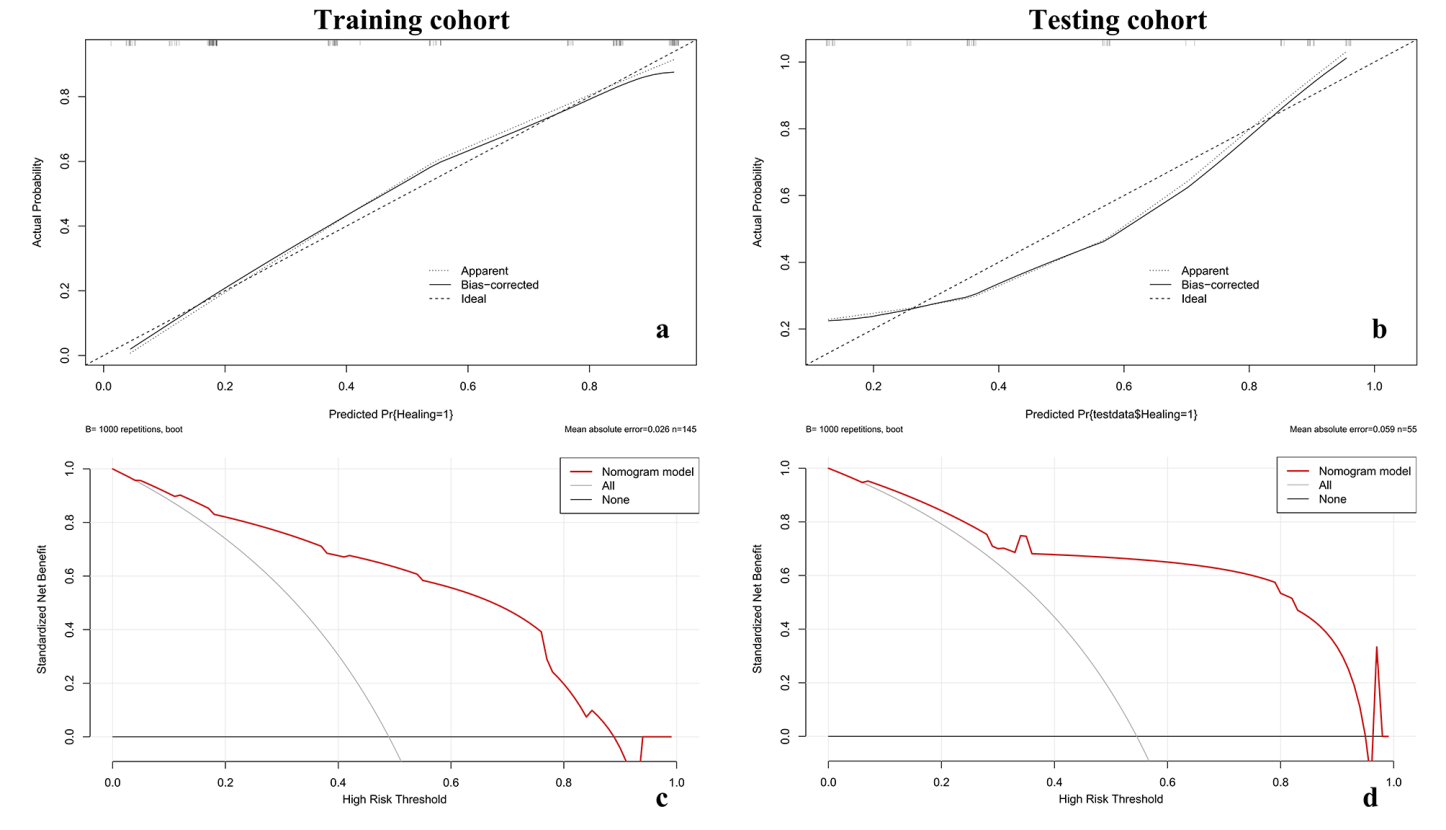
The calibration plots and decision curves of the nomogram model. **a**. The calibration plot in the training cohort. **b**. The calibration plot in the testing cohort. **c**. The decision curve in the training cohort. **d**. The decision curve in the testing cohorts

Figure 5 showed the AUC value of the models in the training and testing cohorts. The AUC value was 0.880 and 0.847 in the training and testing cohorts. Figure 6 showed the calibration and decision curves of the nomogram model for the training and testing cohorts. The calibration curve showed a good correction effect in both cohorts. The decision curve showed that the nomogram may be a useful model for clinical use.

## Discussion

To the best of our knowledge, this is the first study to use a nomogram model to predict early postoperative fistula healing. Our findings demonstrate the significant influence of factors such as CNR value, ADC value, alcohol intake history, and suprasphincter fistula on the healing process. After developing and validating the nomogram model in both the training and testing cohorts, we observed strong predictive capability. Thus, we believe that this innovative model has great potential for clinical applications and can provide valuable insights.

MRI has become the technique of choice for imaging anal fistulas because of its ability to identify tracts, define complex anatomies, and detect abscesses [20, 21]. However, most studies have focused primarily on the utility of preoperative MRI in the assessment of anal fistulas. Recently, in a study conducted by Garg, postoperative MRI was used to evaluate the long-term healing and complications of complex fistulas, demonstrating its high accuracy in assessing healing and post-surgical complications [6]. However, MRI performed shortly after surgery may not adequately distinguish between the postoperative inflammatory granulation and persistent fistula activity. In such cases, both the clinical conditions may appear hyperintense on T2W or STIR sequencing, potentially directing to misleading results. While the traditional approach to assessing MRI examinations for anal fistulas involves qualitative interpretation of signal characteristics, emerging techniques allow for the objective measurement of quantitative parameters that may be indicative of fistula activity [7]. A previous study evaluated the ability of DWI MRI to detect active inflammatory diseases through the measurement of ADC values in the fistula tract. The ADC value reflects the extent of water molecule diffusion through tissues [22]. Boruah et al [23] found that DWI with mean ADC calculation had a good performance in differentiating active from the inactive fistulas. Yoshizako et al [24] also proposed that ADC value of DWI is a feasible method for evaluating perianal fistula activity. Our results are consistent with these findings, suggesting that ADC value of healing group is higher than non-healing, and ADC parameter is a significant factor with anal fistula healing by univariate and multivariate logistic regression analysis.

Recently, several studies have evaluated fistula activity using DCE-MRI, with a particular focus on assessing activity in Crohn’s fistulas [9, 10]. LeFrançois et al. [7] combined DWI and DCE-MRI to differentiate active and inactive anal fistulas. Their findings showed that the transient enhancement observed in DCE-MRI measurements improved the assessment of anal fistula activity, suggesting the potential advantages of incorporating DCE sequences for evaluating anal fistula activity. CNR is defined as the ratio of the absolute value of the signal difference between two tissues to the background noise. This represents the relative difference in signal intensity between the two tissues. Previous studies have shown that DCE-CNR values can be used to effectively classify different tissue classes [25]. Generally, the factors that influence CNR are the inherent differences between tissues and the imaging technology used. In this study, cases were selected based on the same sequence and scanning parameters using DCE sequence for measurements across different cases. Consequently, the differences in the CNR values of the actual measured images primarily reflect the inherent differences between tissues. Active fistulas, characterized by pus and granulation tissue, exhibit high signal intensity on the DCE sequence because of the abundant blood vessels in the granulation tissue. This leads to a higher signal intensity compared with the surrounding muscle tissue, resulting in a higher CNR value. Our results showed that the CNR value of the DCE sequence had sufficient sensitivity and high specificity for assessing early-stage fistula healing.

In addition, it is necessary to understand why postoperative MRI examination is required in patients with anal fistulas. In the present study, almost 54.5% of the patients had horseshoe fistulas and 35.5% of the cohort had recurrent fistulas. For these patients, MR imaging can help doctors and patients understand whether the fistula surgery succeed. Occasionally, although the lesion was adequately treated in the first-stage procedure, after a period of seton treatment, there were cases of poor drainage or even non-healing of the fistula branches or around the posterior portion of the seton. In this circumstance, the second-stage procedure not only needs to incise the position of the seton, but also needs to drainage these places to allow them to heal better.

In our study, we observed significant associations between alcohol intake history, and fistula healing. Alcohol consumption can negatively impact wound healing by affecting both the quality and speed of the healing process [26, 27]. A systematic review has reported an increased risk of postoperative complications, including wound complications, in patients with a history of preoperative alcohol consumption [28]. This could be attributed to the potential reduction in the vascular endothelial growth factor caused by alcohol, which led to a decrease in the formation of new blood vessels.

The main risk factors affecting fistula healing are the type of fistula, number of fistula tracts, and height or location of the internal opening. Previous studies have also reported a higher risk of complications and recurrence of complex fistulas, especially suprasphincteric and extrasphincteric fistulas [29, 30]. Consistent with these reports our results revealed a significant association between suprasphincteric fistulas and failure of healing. A suprasphincteric fistula can be seen as an indication of increased complexity of the fistula [31].

This study has several limitations. First, our study was retrospective and conducted at a single center, which means that it was limited in terms of methodology. To overcome this limitation, a multicenter study should be performed to assess the intra- and inter-observer agreement of measurable MRI parameters and to determine the sensitivity and specificity of different MR technologies in diagnosing fistula healing. Second, the retrospective nature of the study limited the ability to establish a definitive relationship between the amount of alcohol intake and anal fistula healing. Future prospective studies with more comprehensive assessments of alcohol intake as an ordinal variable may provide a more accurate understanding of the association between alcohol intake and fistula healing. Finally, the relatively small sample size may have affected the accuracy of the model used. Therefore, it is important to conduct larger studies to improve the validity and generalizability of our findings.

## Conclusions

We developed a prognostic model and nomogram for predicting fistula healing and achieved good discrimination calibration. This may allow for the prediction of fistula healing in the early postoperative period and could help in the clinical management of anal fistulas.

## Data Availability

All analysed data are included in this published article. The original data are available upon reasonable request from the corresponding author.

## List of abbreviations

BMI: body mass index
CI: confidence interval
ROC: receiver operating characteristic
AUC: area under the curve
ADC: apparent diffusion coefficient
STIR: short tau inversion recovery
DWI: diffusion-weighted imaging
DCE: dynamic contrast-enhanced
IBD: inflammatory bowel disease
EAS: external anal sphincter
ROI: region of interest
CNR: contrast-to-noise ratio
MRI: magnetic resonance imaging
OR: odds ratio
SI: signal intensity
LAVA: liver acquisition with volume acceleration
